# SciFinder

**DOI:** 10.5195/jmla.2018.515

**Published:** 2018-10-01

**Authors:** Stephen Walter Gabrielson

**Affiliations:** M.B. Ketchum Memorial Library, Marshall B. Ketchum University, Fullerton, CA

## INTRODUCTION

SciFinder, a resource from the Chemical Abstracts Service (CAS), is a curated database of chemical and bibliographic information that covers several scientific and biomedical fields, with an emphasis on chemistry. Originally launched in 1995 as a desktop software application, the web version of SciFinder was released in 2008 [1]. According to CAS, the intended audience for SciFinder includes researchers who are affiliated with pharmaceutical companies, universities, and other health sciences settings [2]. SciFinder is an appropriate resource to consult for literature searches and to find background information on chemicals, drugs, and substances.

## CONTENT

SciFinder contains a wide variety of content from journal articles to information on chemical structures, properties, and reactions. There are three separate search sections in SciFinder: references, substances, and reactions.

The bibliographic records that are retrieved from a reference search come from the CAplus and MEDLINE databases. CAplus, produced by CAS, contains journal articles, patents, books, clinical trials, preprints, editorials, and more. It currently contains over 47 million records from more than 180 countries [3] and indexes more than 50,000 journals, 1,500 of which are core chemistry journals whose article records are added to SciFinder within 7 days of publication [4]. Coverage also includes patents from 9 patent offices worldwide, whose records are added 2 days after publication [5]. The majority of CAplus records begin in 1907 and continue to the present, but there are also more than 224,000 patent and article records dating back to 1808 [6].

Records that are retrieved in SciFinder’s substance search section come from the CAS REGISTRY, which contains more than 142 million chemical substances dating back to the early 1800s and is updated on a daily basis [7]. Content in substance records includes property information such as molecular weight, boiling point, spectra, and chemical structure, as well as commercial sources of the substance.

The reactions section of SciFinder contains abundant information on chemical reactions that have been discussed in the literature and in patents. Records retrieved from the reactions search are from CASREACT, another database developed by CAS. CASREACT contains tens of millions of single and multistep reactions that date back to 1840 and is updated daily [8].

## FEATURES

SciFinder’s reference search section can sort results by publication date, author name, and title. Results can also be limited by an impressive range of filters that are divided into three tabbed sections: analyze, refine, and categorize. The analyze tab allows users to filter their results into different subsets, such as by author name. If a user selects this option, the system creates a list of authors based upon the search results and displays the number of references that belong to each author. Users can then select an author from that list to remove all other authors from the results. In the refine filter tab, users can limit results by language, publication year, document type, and more. The categorize filter tab gives users the option to select CAS index terms from different subject categories, which can then be applied to the working result set.

Another notable feature is the ability to remove duplicate MEDLINE records from the search results, which can be done in the analyze filter tab or from the tool’s dropdown menu. The analyze and refine filter tabs are also available in the substance and reactions search sections but have different options such as analyze by bioactivity indictors or substance role or refine by isotope-containing substances or metal-containing substances, among others.

When building a search in the reference section, SciFinder automatically maps keywords to synonyms and includes truncation. This can make searching easier for those who are unfamiliar with building complex searches and who might be unsure of the proper use of truncation symbols. Specific synonyms can be searched by placing up to three synonyms behind a term in parenthesis, with each synonym separated by a comma. SciFinder does not use Boolean operators to construct search queries and, instead, recommends the use of prepositions in the search query to help the database identify the main research concepts.

Another useful feature in SciFinder’s reference search is the citing references tool, which shows how many times a reference has been cited by another paper. Citing references can be found by clicking on the citing references icon next to a reference record on the results page or from within an individual reference record itself. From the individual reference record, users can also find papers that were cited by that reference. This is a great feature, but it should be noted that it lacks the comprehensive citation reports that other databases offer and, thus, should not be the only resource used for evaluating research impact.

There are also features available for saving and returning to specific records from a reference search. One method is to add tags to reference records to find again later. References can also be exported individually or in batches to file types such as RIS format and text files for citation management tools or to portable document format (PDF) and rich text files for other uses. SciFinder can also save answer sets by using the “save” option in the toolbar at the top of the page. Saved searches can be accessed later from the user profile, or SciFinder can generate a uniform resource locator (URL) to be used as a bookmark or to share with others. Alerts to new references added to SciFinder can be created by setting up a Keep Me Posted alert, which is also available in the substance search section of SciFinder.

The three separate search sections in SciFinder are often interoperable. If users find a record from the reference search and are interested in looking up more information about the substances discussed in that article, “Get Substances” can be selected from the reference record to have SciFinder pull up relevant substance records. This also works the other way around, as users can select “Get References” in a substance record to find relevant articles that discuss that particular substance. This feature saves time by avoiding cumbersome navigation between SciFinder’s different search sections.

Users can also search the substance and reactions sections directly, if there is a specific substance or reaction that they wish to find. Records in the substance section can be found using different search options; however, a feature that stands out is the ability to search by using SciFinder’s ChemDraw. Users can draw atoms and bonds using this drag-and-drop tool to find chemical structures or substructures. Structure files from existing SciFinder records can be imported into ChemDraw, which can be useful to share with others for a quick structure look-up.

Lastly, SciFinder features a tool called SciPlanner that provides users with an interactive workspace to help organize and visualize their research. Users can send references, substances, and reactions from all of SciFinder’s databases to their SciPlanner workspace. Here, they can arrange, manipulate, and combine with other reactions to create new reaction pathways. SciPlanner’s drag-and-drop features make completing these tasks simple. Users can share their SciPlanner workspaces by saving them as a PDF document that visualizes the workspace arrangement and includes further information about the reactions, substances, and references that are displayed.

## USABILITY

Because SciFinder is quite comprehensive, it does have a higher learning curve than other databases. Constructing an effective query in the reference search section is not necessarily intuitive to those who are familiar with using Boolean operators in other bibliographic databases. Search queries are instead constructed using prepositions that link concepts together. Despite this, once users learn how to build a search and become accustomed to SciFinder’s layout, they will have little difficulty in accessing content and tools.

Usability is increased by the inclusion of a navigation path, or breadcrumb trail, to show each step of the search process. This assists in keeping track of the search history with ease, while giving users the ability to rerun previous result sets.

SciFinder may feel intimidating initially, but CAS offers extensive training and support material, including short tutorial videos, PDF slides and workbooks with examples, and an extensive help guide that is linked throughout [9].

## COMPARISON TO OTHER RESOURCES

SciFinder overlaps with other resources in that it includes MEDLINE records, has citing reference features, and contains chemical substance information that users can find for free through National Library of Medicine resources. However, SciFinder’s coverage of the biomedical sciences and its strong focus on chemical information in the literature sets this resource apart from other bibliographic databases. Also, the millions of substance and reaction records found in SciFinder surpass the amount of content from other drug and chemical information resources.

One area that SciFinder lags behind is in the lack of more specific publication-type filters to help narrow results down to the strongest evidence in the literature, such as systematic reviews and meta-analyses. To work around this, users need to include specific study types as search terms in the query.

## CONCLUSION

SciFinder is a robust, curated bibliographic database that also includes an impressive amount of chemical records and provides many features that help users find information and manage their research. While librarians may find aspects of this resource useful for tasks such as measuring research impact, patrons would get the most use out of this resource in accomplishing a wide range of research queries. Despite the time investment of learning how to effectively use SciFinder, it is a worthwhile resource geared toward library patrons who study and work in chemistry, pharmacy, engineering, and other health sciences fields.
